# AptaNet as a deep learning approach for aptamer–protein interaction prediction

**DOI:** 10.1038/s41598-021-85629-0

**Published:** 2021-03-16

**Authors:** Neda Emami, Reza Ferdousi

**Affiliations:** 1 Department of Health Information Technology, School of Management and Medical Informatics, Tabriz University of Medical Sciences, Tabriz, Iran; 2 Research Center for Pharmaceutical Nanotechnology, Biomedicine Institute, Tabriz University of Medical Sciences, Tabriz, Iran

**Keywords:** Computational biology and bioinformatics, Mathematics and computing, Biological techniques

## Abstract

Aptamers are short oligonucleotides (DNA/RNA) or peptide molecules that can selectively bind to their specific targets with high specificity and affinity. As a powerful new class of amino acid ligands, aptamers have high potentials in biosensing, therapeutic, and diagnostic fields. Here, we present AptaNet—a new deep neural network—to predict the aptamer–protein interaction pairs by integrating features derived from both aptamers and the target proteins. Aptamers were encoded by using two different strategies, including k-mer and reverse complement k-mer frequency. Amino acid composition (AAC) and pseudo amino acid composition (PseAAC) were applied to represent target information using 24 physicochemical and conformational properties of the proteins. To handle the imbalance problem in the data, we applied a neighborhood cleaning algorithm. The predictor was constructed based on a deep neural network, and optimal features were selected using the random forest algorithm. As a result, 99.79% accuracy was achieved for the training dataset, and 91.38% accuracy was obtained for the testing dataset. AptaNet achieved high performance on our constructed aptamer-protein benchmark dataset. The results indicate that AptaNet can help identify novel aptamer–protein interacting pairs and build more-efficient insights into the relationship between aptamers and proteins. Our benchmark dataset and the source codes for AptaNet are available in: https://github.com/nedaemami/AptaNet.

## Introduction

In 1990, aptamers were first introduced in the last decade of the twentieth^1–3^. They are short single-stranded sequences (RNA/DNA) or peptides. Because of their spatial conformations, they are capable of binding to specific molecular targets with high specificity and affinity^4^. These biological targets can be a broad range of biomolecules (e.g., lipids^5^, viruses^6^, nucleic acids^7^, cytokines^8^, ions^9^, etc.).

Aptamers are analogous to antibodies, but they have more advantages that make them a better choice. First, the aptamers are more stable at high temperatures. Second, after selection, aptamers amplify easily by polymerase chain reactions to establish large amounts of molecules with high purity. Third, aptamers are screened by the in-vitro process using an artificial library instead of cell lines or animals. And finally, aptamers have a simple structure, so they could be easily modified by adding various functional groups^10–12^. Therefore, as a new class of amino acid ligands, aptamers are expected to have great potential in bio sensing, diagnostics, and therapeutic approaches.

Aptamers are selected and produced by an in-vitro process, called the systematic evolution of ligands by exponential enrichment (SELEX), which contains a repetitive cycle of selection and amplification^3,13^. The process of empirical SELEX is challenging, time-consuming, and often fails to enrich high-affinity aptamers^14,15^. So far, several efforts have been conducted to improve the production and selection of aptamers^16^. However, it is still necessary for to develop other computational methods to accelerate the process, save cost, and design more effective aptamers with high affinity and specificity.

According to the best of our knowledge, four computational methods have been developed so far in terms of predicting aptamer–protein interaction. For the first time, Li et al.^17^ proposed a predictor based on a random forest (RF) algorithm. They used nucleotide composition for encoding aptamers, and targets were encoded using AAC and PseAAC. Although their predictor had yielded favorable results, but it had an imbalance problem. Zhang and co-workers^18^ developed an ensemble classifier consisting of three RF sub-classifiers to deal with the imbalance problem. They used pseudo K-tuple nucleotide composition (PseKNC) to represent aptamers and discrete cosine transform, bigram position-specific scoring matrix, and disorder information used for target encoding. In another work for imbalanced data handling, in 2019, Yang et al.^19^ presented an ensemble classifier with three support vector machine (SVM)s sub-classifiers. They used nucleic acid composition and PseKNC for aptamer encoding, and a sparse autoencoder was applied to represent the targets. And recently, Li et al.^20^ developed a web server to predict protein–aptamer interactions using an integrated framework Adaboost and random forest and features derived from sequences of aptamers and proteins. Aptamers were represented by nucleotide composition, PseKNC, and normalized Moreau-Broto autocorrelation coefficient. However, proteins were characterized by AAC, PseAAC, grouped amino acid composition, C/T/D composition and sequence-order-coupling number.

These four methods have been developed to generate interaction predictions and yielded good results, but there are several opportunities and requirements for enhancing this field. Since deep learning techniques have had a high performance in biological predictions^21–24^, and they have not yet been exploited as a predictor to predict aptamer–protein interactions (API). Therefore, in this paper, we present AptaNet (a first deep neural network predictor) to predict the API pairs by integrating features derived from both aptamers and the target proteins. Aptamers were encoded by using two different strategies includes k-mer and revck-mer frequency. AAC and PseAAC were applied to represent target information using 24 physicochemical and conformational properties of proteins. To handle the imbalance problem in our dataset, we applied neighborhood cleaning algorithm.

AptaNet achieved high performance on our constructed aptamer-protein benchmark dataset. The results indicate that AptaNet could help to identify novel aptamer–protein interacting pairs and build more-efficient biological insights into understanding the relationship between aptamers and proteins, which could benefit all aptamer scientists and researchers.

The rest of the paper is organized as follows: presentation of the predictor's performance using cross-validation, discussion, conclusion, dataset description, statistical samples formulation, selection, and development of a deep neural network.

## Results

The technical details of AptaNet are provided in the “Materials and Methods” section. In this section, the results of several evaluation experiments are presented.

### The results of feature group effects

We created four groups (1, 2, 3, and 4). Each group consists of eight feature groups to describe aptamers encoding by the K-mer (k = 3), K-mer (k = 4), RevcK-mer (k = 3), and RevcK-mer (k = 4) methods, respectively, with proteins that encoded by AAC and PseAAC. We used a total of 24 properties (i.e., physicochemical, conformational, and energetic) of proteins. We added three groups of properties each time to the previous dataset, sequentially. The feature groups and their information have been reported in Table 1. We have performed four sets of experiments to investigate the feature group's effectiveness. In these experiments, we changed the feature groups on different deep neural networks by applying a balancing method to analyze the effect of features and performance of different neural networks. The results of these experiments are reported in Table 2. It is noticeable that the best results are related to the balanced datasets.

**Table 1:** Feature group description.

Group	Aptamer encoding			Protein encoding			Dataset	Total number
	Method	Group name	Number of feature	Features	Group name	Number of feature		
Group 1	Kmer (k = 3)	Apt	84	hydrophobicity, hydrophilicity, mass	A	50	Apt + A	134
				polarity, molecular weight, melting point	B	50	Apt + A + B	184
				transfer free energy, buriability, bulkiness	C	50	Apt + A + B + C	234
				solvation free energy, relative mutability, residue volume	D	50	Apt + A + B + C + D	384
				volume, amino acid distribution, hydration number	E	50	Apt + A + B + C + D + E	334
				isoelectric point, compressibility, chromatographic index	F	50	Apt + A + B + C + D + E + F	384
				unfolding entropy change, unfolding enthalpy, unfolding Gibbs free energy charge	G	50	Apt + A + B + C + D + E + F + G	434
				Power of N terminal of alphahelix, Power of C terminal of alphahelix, Power of best the middle of alphahelix	H	50	Apt + A + B + C + D + E + F + G + H	484
Group 2	Kmer (k = 4)	Apt	339	hydrophobicity, hydrophilicity, mass	A	50	Apt + A	389
				polarity, molecular weight, melting point	B	50	Apt + A + B	439
				transfer free energy, buriability, bulkiness	C	50	Apt + A + B + C	489
				solvation free energy, relative mutability, residue volume	D	50	Apt + A + B + C + D	539
				volume, amino acid distribution, hydration number	E	50	Apt + A + B + C + D + E	589
				isoelectric point, compressibility, chromatographic index	F	50	Apt + A + B + C + D + E + F	639
				unfolding entropy change, unfolding enthalpy, unfolding Gibbs free energy charge	G	50	Apt + A + B + C + D + E + F + G	689
				Power of N terminal of alphahelix, Power of C terminal of alphahelix, Power of best the middle of alphahelix	H	50	Apt + A + B + C + D + E + F + G + H	739
Group 3	Revckmer (k = 3)	Apt	44	hydrophobicity, hydrophilicity, mass	A	50	Apt + A	94
				polarity, molecular weight, melting point	B	50	Apt + A + B	144
				transfer free energy, buriability, bulkiness	C	50	Apt + A + B + C	194
				solvation free energy, relative mutability, residue volume	D	50	Apt + A + B + C + D	244
				volume, amino acid distribution, hydration number	E	50	Apt + A + B + C + D + E	294
				isoelectric point, compressibility, chromatographic index	F	50	Apt + A + B + C + D + E + F	344
				unfolding entropy change, unfolding enthalpy, unfolding Gibbs free energy charge	G	50	Apt + A + B + C + D + E + F + G	394
				Power of N terminal of alphahelix, Power of C terminal of alphahelix, Power of best the middle of alphahelix	H	50	Apt + A + B + C + D + E + F + G + H	444
Group 4	Revckmer (k = 4)	Apt	179	hydrophobicity, hydrophilicity, mass	A	50	Apt + A	229
				polarity, molecular weight, melting point	B	50	Apt + A + B	279
				transfer free energy, buriability, bulkiness	C	50	Apt + A + B + C	329
				solvation free energy, relative mutability, residue volume	D	50	Apt + A + B + C + D	379
				volume, amino acid distribution, hydration number	E	50	Apt + A + B + C + D + E	429
				isoelectric point, compressibility, chromatographic index	F	50	Apt + A + B + C + D + E +	479
				unfolding entropy change, unfolding enthalpy, unfolding Gibbs free energy charge	G	50	Apt + A + B + C + D + E + F + G	529
				Power of N terminal of alphahelix, Power of C terminal of alphahelix, Power of best the middle of alphahelix	H	50	Apt + A + B + C + D + E + F + G + H	579

Where Kmer (k = 3) is 3mer frequency, Kmer (k = 4) is 4mer frequency, Revckmer (k = 3) is reverse complement 3mer, and Revckmer frequency (k = 4) is reverse complement 4mer frequency for apt, which represents aptamer properties. And, Apt indicates aptamer properties.For protein properties: A indicates hydrophobicity, hydrophilicity, and mass; B indicates polarity, molecular weight, and melting point; C indicates transfer free energy, buriability, and bulkiness; D indicates solvation free energy, relative mutability, and residue volume; E indicates volume, amino acid distribution, and hydration number; F indicates isoelectric point, compressibility, and chromatographic index; G indicates unfolding entropy change, unfolding enthalpy and unfolding Gibbs free energy change; H indicates power to beat the N terminal, C terminal, and middle of the alpha helix.

**Table 2:** The average performances of two deep neural network classifiers on 32 different datasets, with and without the balancing method.

Dataset	Feature combination	Deep neural networks F1-score (imbalanced)		Deep neural networks F1-score (balanced)	
		MLP	CNN	MLP	CNN
Kmer (k = 3) + PseAAC	Apt + A	0.8071	0.8197	0.8417	0.8577
	Apt + A + B	0.7992	0.8624	0.8507	0.8416
	Apt + A + B + C	0.8002	0.8420	0.8510	0.8465
	Apt + A + B + C + D	0.7800	0.8409	0.8555	0.8553
	Apt + A + B + C + D + E	0.7764	0.7406	0.8584	0.7983
	Apt + A + B + C + D + E + F	0.7675	0.7618	0.8539	0.7829
	Apt + A + B + C + D + E + F + G	0.7824	0.8043	0.8598	0.8456
	Apt + A + B + C + D + E + F + G + H	0.7753	0.8455	0.8497	0.8105
Kmer (k = 4) + PseAAC	Apt + A	0.8610	0.8542	0.8739	0.8630
	Apt + A + B	0.8461	0.8551	0.8616	0.8720
	Apt + A + B + C	0.8307	0.8608	0.8521	0.8220
	Apt + A + B + C + D	0.8299	0.8347	0.8660	0.8184
	Apt + A + B + C + D + E	0.7997	0.8086	0.8410	0.8379
	Apt + A + B + C + D + E + F	0.7908	0.8389	0.8761	0.8354
	Apt + A + B + C + D + E + F + G	0.8173	0.8257	0.8493	0.8038
	Apt + A + B + C + D + E + F + G + H	0.8130	0.8228	0.8595	0.7939
Revckmer (k = 3) + PseAAC	Apt + A	0.7689	0.8196	0.8215	0.8564
	Apt + A + B	0.7562	0.8287	0.8183	0.8567
	Apt + A + B + C	0.7596	0.8210	0.8119	0.8513
	Apt + A + B + C + D	0.7411	0.8074	0.8265	0.8240
	Apt + A + B + C + D + E	0.7532	0.8418	0.8203	0.8349
	Apt + A + B + C + D + E + F	0.7302	0.8364	0.8256	0.8517
	Apt + A + B + C + D + E + F + G	0.7321	0.7150	0.8192	0.7990
	Apt + A + B + C + D + E + F + G + H	0.7347	0.8043	0.8246	0.8368
Revckmer (k = 4) + PseAAC	Apt + A	0.7870	0.8524	0.8471	0.8559
	Apt + A + B	0.8192	0.8125	0.8434	0.8416
	Apt + A + B + C	0.8020	0.8566	0.8542	0.8537
	Apt + A + B + C + D	0.7928	0.8560	0.8581	0.8501
	Apt + A + B + C + D + E	0.7846	0.8278	0.8378	0.8317
	Apt + A + B + C + D + E + F	0.7893	0.8325	0.8332	0.8168
	Apt + A + B + C + D + E + F + G	0.7839	0.8288	0.8404	0.7995
	Apt + A + B + C + D + E + F + G + H	0.7725	0.7971	0.8393	0.8195

Where Kmer 3 is 3mer frequency, Kmer 4 is 4mer frequency, Revckmer 3 is reverse complement 3mer, and Revckmer frequency 4 is reverse complement 4mer frequency for apt, which represents aptamer properties. For protein properties: A indicates hydrophobicity, hydrophilicity, and mass; B indicates polarity, molecular weight, and melting point; C indicates transfer free energy, buriability, and bulkiness; D indicates solvation free energy, relative mutability, and residue volume; E indicates volume, amino acid distribution, and hydration number; F indicates isoelectric point, compressibility, and chromatographic index; G indicates unfolding entropy change, unfolding enthalpy and unfolding Gibbs free energy change; H indicates power to beat the N terminal, C terminal, and middle of the alpha helix. *MLP* multi-layer perceptron; *CNN* convolutional neural network.

Since the number of properties is high, we have briefly named every three groups of properties: A, B, C, D, E, F, G, and H. A: hydrophobicity, hydrophilicity, mass; B: polarity, molecular weight, melting point; C: transfer free energy, buriability, bulkiness; D: solvation free energy, relative mutability, residue volume; E: volume, amino acid distribution, hydration number; F: isoelectric point, compressibility, chromatographic index; G: unfolding entropy change, unfolding enthalpy, unfolding Gibbs free energy change; H: the power to beat the N terminal, C terminal, and middle of alpha-helix; and apt for aptamers. It should be noted that various combinations of these 24 properties were examined (e.g., hydrophobicity, hydrophilicity, mass; hydrophobicity, hydrophilicity, polarity; isoelectric point, compressibility, hydrophilicity, etc.), and finally, the best combinations were selected based on their results.

First, we evaluate the performance using feature group k-mer2 + A, next added A + B, A + B + C, and finally A + B + C + D + E + F + G + H, sequentially. We have generated the average results of each feature group and different combinations of these feature groups based on sequential forward selection.

Table 2 represents the average performance of two different neural networks on the 32 datasets during our experiments. We have generated the results of various combinations of the feature groups by adding them in a forward selection scheme by sorting them based on their spatial performance for different aptamer encoding methods. For k-mer = *4*, the best results were achieved when 21 properties(i.e., hydrophobicity, hydrophilicity, mass, polarity, molecular weight, melting point, transfer-free energy, buriability, bulkiness, solvation free energy, relative mutability, residue volume, volume, amino acid distribution, hydration number, isoelectric point, compressibility, chromatographic index, unfolding entropy change, unfolding enthalpy, and unfolding Gibbs free energy charge) were applied. In the second place, the combination of 15 properties (i.e., hydrophobicity, hydrophilicity, mass, polarity, molecular weight, melting point, transfer-free energy, buriability, bulkiness, solvation free energy, relative mutability, residue volume, volume, amino acid distribution, and hydration number) had the highest performance.

For k-mer = *3,* the best results were achieved when 18 properties (i.e., hydrophobicity, hydrophilicity, mass, polarity, molecular weight, melting point, transfer-free energy, buriability, bulkiness, solvation free energy, relative mutability, residue volume, volume, amino acid distribution, hydration number, isoelectric point, compressibility, and chromatographic index) were applied. In the second place, the combination of 12 properties (i.e., hydrophobicity, hydrophilicity, mass, polarity, molecular weight, melting point, transfer-free energy, buriability, bulkiness, solvation free energy, relative mutability, and residue volume) had the highest performance.

For the Revck-mer = *3*, the best results were achieved when six properties (i.e., hydrophobicity, hydrophilicity, mass, polarity, molecular weight, and melting point) were applied. In the second place, the combination of three properties (i.e., hydrophobicity, hydrophilicity, and mass) had the highest performance.

For the Revck-mer = *4*, the best results were achieved when 12 properties (i.e., hydrophobicity, hydrophilicity, mass, polarity, molecular weight, melting point, transfer-free energy, buriability, bulkiness, solvation free energy, relative mutability, and residue volume) were used. In the second place, the combination of 9 properties (i.e., hydrophobicity, hydrophilicity, mass, polarity, molecular weight, melting point, transfer-free energy, buriability, and bulkiness). had the highest performance.

Therefore, according to the results of *32* different datasets, four datasets were selected which had the best values in each group (i.e., Apt + A + B + C + D + E + F + G in group 1, Apt + A + B + C + D + E + F in group 2, Apt + A + B in group 3, and Apt + A + B + C + D in group 4).

### The results of neural networks performances

To test and select the appropriate deep neural network for our problem, we tested two deep neural networks: MLP and CNN. The experiment was performed once on the balance data and once on the imbalance data. For these experiments, we applied random under-sampling as the balancing method. Thirty-two different combinations were used as features that have been mentioned already. To set the neural networks: the number of batch sizes for MLP and CNN were 310 and 16, respectively. Additionally, the number of epochs was determined 200, rmsprop was also considered as an optimizer with its default values, and the activation function was sigmoid. The results regarding the F1-score were presented in Table 2.

In Table 2, the highest performance values achieved from MLP and CNN are highlighted. It is evident that MLP provides the highest values for 22 different feature group combinations. And CNN has achieved the highest values for ten datasets.

Except for three cases, including Apt + A, Apt + A + B, and Apt + A, MLP have the highest values in the three groups 1, 2, and 4. Also exception of Apt + A + B + C + D and Apt + A + B + C + D + E + F + G, CNN has the highest values in group 3. It can be deduced, that by decreasing the dimensionality of datasets CNN has better performance. In the other words, MLP achieves better performance for datasets with more dimension. Therefore, we select MLP as out classifier.

### The results of MLP with machine learning algorithms

**Figure 1:**
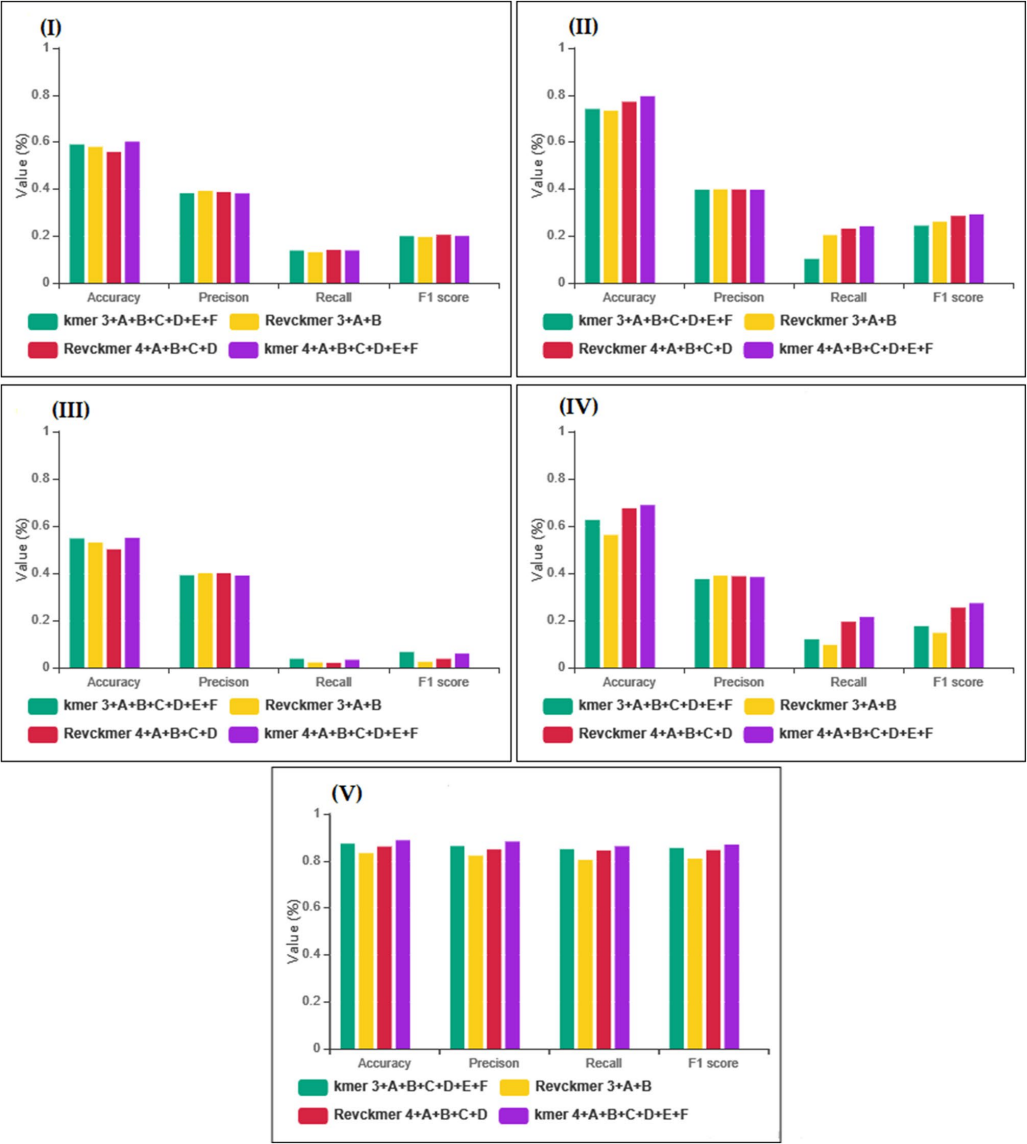
The comparison of the prediction performance of our model and four machine learning algorithms on four different datasets. Where Kmer 3 is 3mer frequency, Kmer 4 is 4mer frequency, Revckmer 3 is reverse complement 3mer, and Revckmer frequency 4 is reverse complement 4mer frequency for aptamer properties. For protein properties: A indicates hydrophobicity, hydrophilicity, and mass; B indicates polarity, molecular weight, and melting point; C indicates transfer free energy, buriability, and bulkiness; D indicates solvation free energy, relative mutability, and residue volume; E indicates volume, amino acid distribution, and hydration number; F indicates isoelectric point, compressibility, and chromatographic index. (**I**) is results of k nearest neighbor algorithm; (**II**) is results of random forest algorithm; (**III**) is results of support vector machine algorithm; (**IV)** is results shallow neural network algorithm and (**V**) is results of our model. The power of predictors is calculated based on four metrics, including accuracy, precision, recall, and F1score.

In this section, we compared the performance of AptaNet against some machine learning algorithms (shallow neural networks (SNN), k-nearest neighbor (KNN), RF, and SVM). All of algorithms were implemented in SKlearn library with the following parameters: Knn: n = 5, leaf size = 25, p = 2; RF: max depth = 3, n estimators = 10; SVM: degree = 3, c = 1, kernel = 'linear', probability = True, cache size = 200; SNN: hidden layer = (3, 2); solver = 'lbfgs', alpha = 0.0001. Which, all parameters were determined by different experiments. We performed a fivefold cross-validation strategy to evaluate the results of the test and training set. The average performance results are indicated in Table 3 and Fig. 1.

**Table 3:** The average performances of our model with four machine learning algorithms on four different datasets.

Datasets	Methods	Accuracy	Precision	F1 Score	Matthews's correlation coefficient	Specificity	Sensitivity
Kmer 3 + A + B + C + D + E + F	Shallow neural network	0.625	0.374	0.175	0.019	0.894	0.118
	K nearest neighbor	0.589	0.381	0.199	0.002	0.864	0.137
	Random forest	0.739	0.397	0.242	0.13	0.914	0.181
	Support vector machine	0.546	0.39	0.065	− 0.022	0.954	0.036
	Multilayer perceptron	0.872	0.861	0.854	0.741	0.89	0.849
Kmer 4 + A + B + C + D + E + F	Shallow neural network	0.687	0.382	0.272	0.101	0.868	0.212
	K nearest neighbor	0.601	0.38	0.2	0.011	0.871	0.136
	Random forest	0.795	0.396	0.291	0.196	0.918	0.24
	Support vector machine	0.548	0.39	0.058	− 0.02	0.96	0.032
	Multilayer perceptron	0.886	0.881	0.869	0.77	0.906	0.861
Revckmer_3 + A + B	Shallow neural network	0.561	0.388	0.146	− 0.018	0.892	0.095
	K nearest neighbor	0.578	0.391	0.194	− 0.001	0.868	0.13
	Random forest	0.732	0.398	0.259	0.136	0.901	0.203
	Support vector machine	0.529	0.4	0.043	− 0.022	0.97	0.021
	Multilayer perceptron	0.831	0.821	0.809	0.576	0.948	0.558
Revckmer_4 + A + B + C + D	Shallow neural network	0.674	0.386	0.252	0.085	0.873	0.192
	K nearest neighbor	0.557	0.387	0.203	− 0.019	0.846	0.139
	Random forest	0.77	0.397	0.284	0.175	0.909	0.23
	Support vector machine	0.5	0.4	0.036	− 0.029	0.971	0.019
	Multilayer perceptron	0.86	0.847	0.845	0.718	0.874	0.843

Where Kmer 3 is 3mer frequency, Kmer 4 is 4mer frequency, Revckmer 3 is reverse complement 3mer, and Revckmer frequency 4 is reverse complement 4mer frequency for aptamer properties. For protein properties: A indicates hydrophobicity, hydrophilicity, and mass; B indicates polarity, molecular weight, and melting point; C indicates transfer free energy, buriability, and bulkiness; D indicates solvation free energy, relative mutability, and residue volume; E indicates volume, amino acid distribution, and hydration number; F indicates isoelectric point, compressibility, and chromatographic index.

According to Table 3, the highest accuracy belongs to MLP for all datasets. Which among them, the best performance was obtained when kmer4_apt + A + B + C + D + E + F were applied.

In second place among the machine learning algorithms, RF has the highest accuracy for all four datasets. The best performance was obtained using kmer4_apt + A + B + C + D + E + F, which could be due to RF as an ensemble classifier consisting of multiple decision trees. Since RF has less overfitting to a particular dataset, accuracy was better than other machine learning algorithms. Therefore, according to four different datasets' accuracy values, kmer4_apt + A + B + C + D + E + F was selected as a dataset for our model (See Supplementary Table S1).

Among the machine learning algorithms, the lowest performance was achieved for SVM algorithm when aptamer features were combined with 12 properties of protein properties.

The lowest performance among datasets was achieved when aptamer features were combined with six protein properties (i.e., hydrophobicity, hydrophilicity, mass, polarity, molecular weight, melting point).

### The results of the MLP optimization and feature selection

Since MLP had the highest performance among other deep learning and machine learning methods, MLP was selected as our predictor model. To improve our model's performance, we implemented it by selecting the most optimal parameters with different experiments and the final model named AptaNet. We optimized our model with different values for the learning rate, batch size, and epochs. Learning rate = 0.00014, batch size = 5000, and epochs = 260 were the final settings of the presented model.

Also, we applied RF strategy for feature selection and ranking features. The optimal number of features was set to 193 by several experiments on RF parameters and different numbers of features. The 193 optimal features were selected according to the nature of our dataset and parameters of the RF strategy. The parameters were set based on our several feature selection experiments. We set estimators = 300 and max depth = 9 based on our feature selection experiments (See Supplementary Table S2).

**Figure 2:**
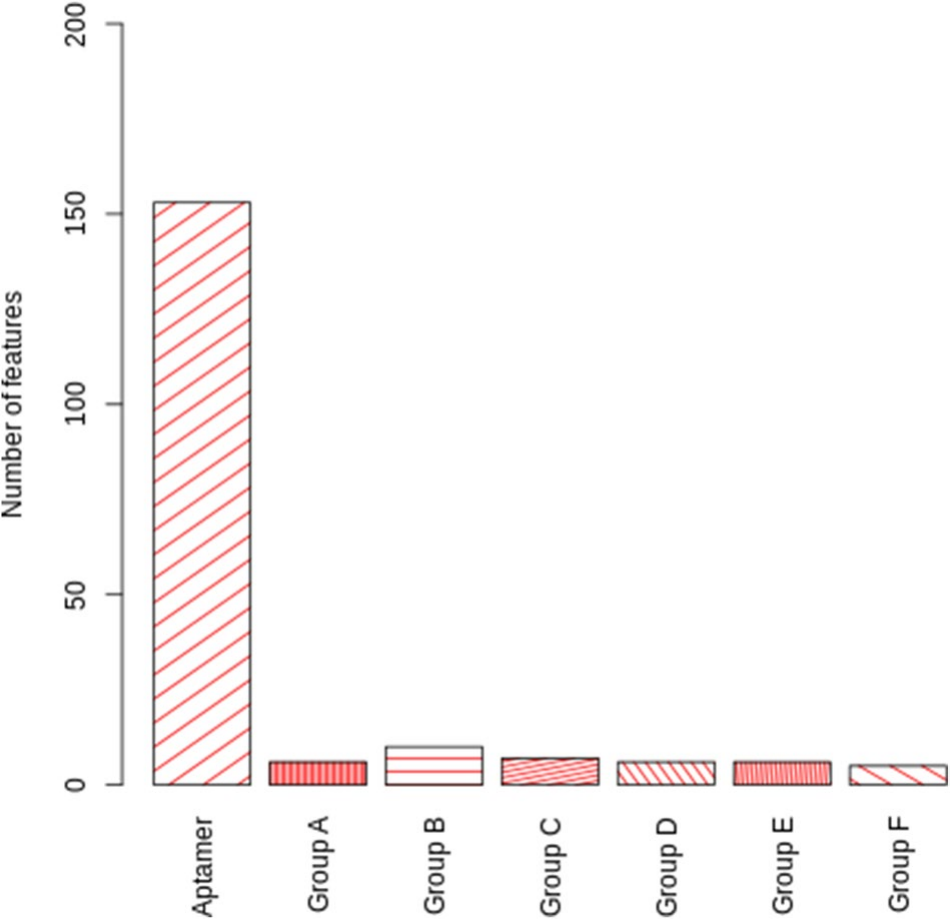
Distributions of the 193 optimal features. Where group A represents hydrophilicity and mass; group B represents polarity, molecular weight, and melting point; group C represents transfer free energy, buriability, and bulkiness; group D represent solvation free energy, relative mutability, and residue volume; group E represent volume, amino acid distribution, and hydration number; group F represent isoelectric point, compressibility and chromatographic index for protein properties.

Figure 2 describes the feature's importance in ranking values.

As shown in Fig. 2, 193 optimal features obtained from RF strategy could be classified into eight terms: k-mer aptamer frequency, protein composition A, protein composition B, and protein composition F.

In the first place, k-mer aptamer frequency ranks the first; making up approximately 73%. In other words, a considerable part of the optimal features belongs to aptamer features. The k-mer aptamer frequency implies that using k-mer is a key factor for API in this study.

In the second place, there is feature composition B. As follows, in the third place, there is feature composition C. In the fourth place, another effective trait is feature composition A, composition D, and E, in which their counts are nearly equal. This finding means that the impact of using these three feature composition is the same. And in the last place, there is feature composition F.

**Figure 3:**
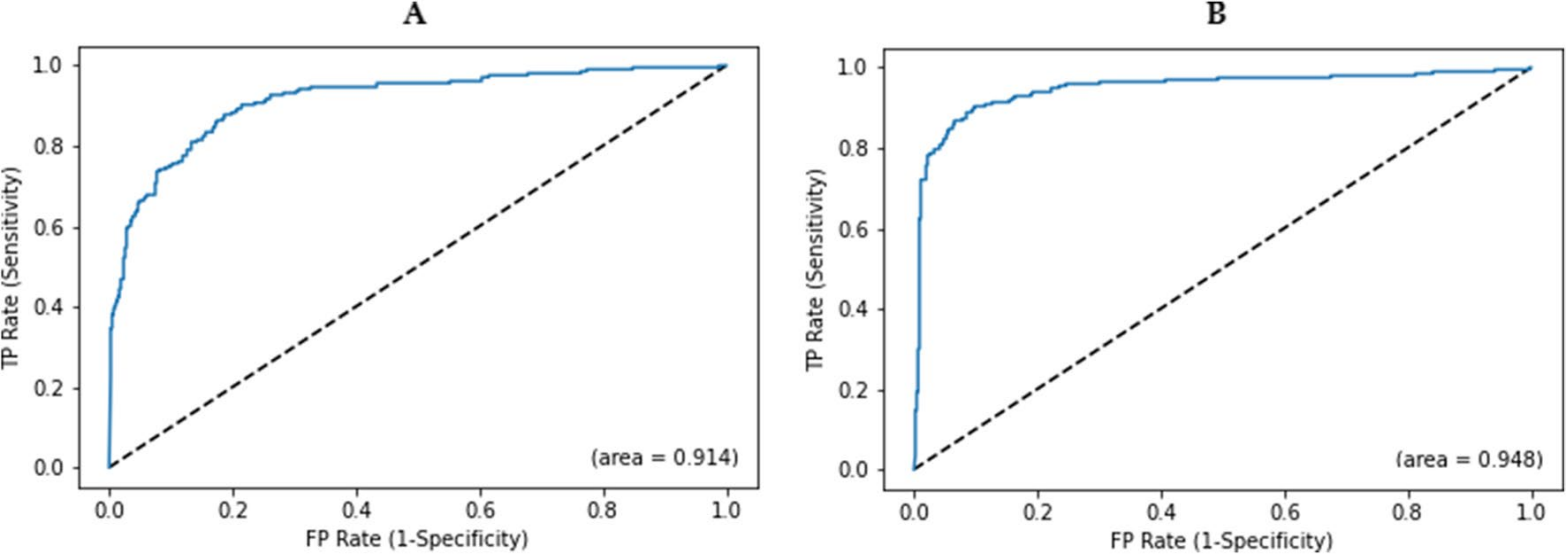
Receiver operating characteristic (ROC) curves of AptaNet before and after the feature selection on our benchmark dataset. Where (**A**) depicts the prediction performance of AptaNet before using feature selection and (**B**) illustrates the prediction performance of AptaNet after using feature selection. *FP* False Positive rate, *TP* True Positive rate.

According to Fig. 3, the ROC value for AptaNet was 0.914 before feature selection and 0.948 after feature selection.

Moreover, the results of testing and training of AptaNet before and after applying feature selection are presented in Tables 4 and 5, respectively. All results of the AptaNet were higher after applying the feature selection technique.

**Table 4:** The overall results of AptaNet before feature selection.

Dataset	Accuracy	Precision	F1-score	Matthews’s correlation coefficient	Specificity	Sensitivity
Kmer 4 + A + B + C + D + E + F (train)	0.998	0.998	0.998	0.997	0.999	0.998
Kmer 4 + A + B + C + D + E + F (test)	0.892	0.883	0.877	0.782	0.908	0.873

Where Kmer 4 is 4mer frequency for aptamer properties. For protein properties: A indicates hydrophobicity, hydrophilicity, and mass; B indicates polarity, molecular weight, and melting point; C indicates transfer free energy, buriability, and bulkiness; D indicates solvation free energy, relative mutability, and residue volume; E indicates volume, amino acid distribution, and hydration number; F indicates isoelectric point, compressibility, and chromatographic index.

**Table 5:** The overall results of AptaNet after feature selection.

Dataset	Accuracy	Precision	F1-score	Matthews’s correlation coefficient	Specificity	Sensitivity
Kmer 4 + A + B + C + D + E + F (train)	0.997	0.997	0.997	0.995	0.995	0.999
Kmer 4 + A + B + C + D + E + F (test)	0.913	0.909	0.899	0.824	0.931	0.89

Where Kmer 4 is 4mer frequency for aptamer properties. For protein properties: A indicates hydrophobicity, hydrophilicity, and mass; B indicates polarity, molecular weight, and melting point; C indicates transfer free energy, buriability, and bulkiness; D indicates solvation free energy, relative mutability, and residue volume; E indicates volume, amino acid distribution, and hydration number; F indicates isoelectric point, compressibility and chromatographic index.

**Figure 4:**
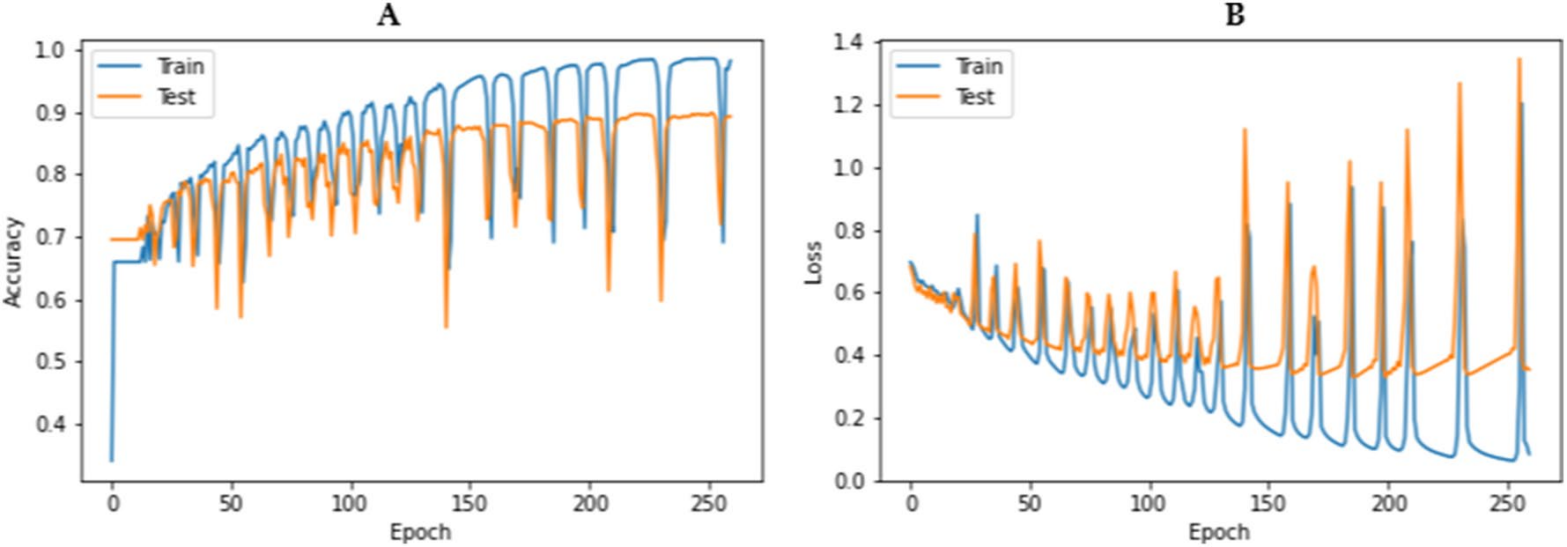
Model accuracy and loss of AptaNet on our benchmark dataset. Where (**A**) is model accuracy, and (**B**) is model loss of AptaNet.

Additionally, Fig. 4 illustrates the model accuracy and loss of AptaNet for epoch = 260 and batch size = 5000.

## Discussion

One of the most important challenges in the field of aptamer–target interaction is that the aptamers' special databases(DBs) are scarce. To the best of our knowledge, there are four DBs about aptamer–target interaction, containing Aptamer Database^25^, RiboaptDB^26^, Aptamer Base^27^, and Aptagen (https://www.aptagen.com/). Which among them, Aptamer Database and RiboaptDB no longer exist. Therefore, in this study, to have a complete dataset of API pairs, for the first time, in addition to Freebase, we also used Aptagen data, which are generated by independent studies. Aptagen provides useful information about aptamer type, target type, and experimental conditions.

Kmer frequency method has been widely used in many bioinformatics studies^28–30^ and has had successful results. The basic idea behind this method is that the encoding of each item is based on its interaction with its context. If we consider each sequence as a sentence and each K-mer as a word, we can extend this method to encode aptamer and protein sequences. Thus, Because of the simplicity, considering almost more sequence information compared to other methods available for encoding nucleotide sequences, we have used this method for encoding aptamer sequences.

One of the main problems in AAC strategy is losing the information of protein sequences. To overcome this restriction that can be affected on prediction performances, we have used PseAAC method. In the previous studies related to protein interaction prediction PseAAC method has been widely used and has had successful outcomes^31–37^. Therefore, we applied this strategy to representing protein target sequences in this study.

In several previous studies, it is proved that the physicochemical properties (e.g., hydrophilicity, hydrophobicity, average accessible surface area, and polarity) and biochemical contacts (e.g., residue contacts, atom contacts, salt bridges, and hydrogen bonds) play an essential and constructive role in protein interactions^38–44^. Thus, we have used 32 structural-based and sequence-based properties from protein sequences. However, in the previous studies related to aptamer–target interaction, this large volume of features has not been used.

In this study, we have used the NCL strategy to overcome the imbalanced dataset problem. According to Table 2, in the experimental results obtained from the two neural networks (MLP and CNN), the results on the test dataset were significantly improved after applying the NCL method. Since the NCL technique not only focuses on data reduction but also focuses on cleaning data. NCL reduces the majority class by eliminating low-quality data using Edited Nearest Neighbor (ENN) rules. Moreover, ENN removes classified data that are classified incorrectly. Therefore, the data cleaning process is intended for both majority and minority class samples^45,46^. Consequently, according to^46–50^, NCL as an under-sampling method has superior outcomes compared to other common over-sampling methods.

As a subfield of machine learning approaches, deep learning methods have been shown to exhibit unprecedented performance in various areas of biological prediction^51–61^. We described a novel deep neural network model in the present study, termed AptaNet, for predicting API.

We compared the MLP and CNN performances on the 32 different datasets to develop our prediction model. The performance of each network and algorithm was determined by assessing how they could correctly predict whether the aptamers were interacting with a specific target or not.

According to Table 2, MLP had superior outcomes compared to CNN. According to^62–68^, MLP networks have been more efficient in text processing problems. In this study, since API data were similar to text data, MLP networks had higher performance. Moreover, CNNs have had better outcomes in classification image data^69–74^.

Next, we compared MLP against some machine learning algorithms. Among the applied machine learning algorithms, the SVM algorithm achieved the lowest performance when aptamer features were combined with 12 protein properties. The lowest performance of the SVM algorithm in the prediction of API may be attributed to the following shortcomings.

According to^75–78^, First, the SVM algorithm generally has not a convenient performance for large data sets. Second, in cases where the dataset has noises (target classes are overlapping), the SVM classifier will underperform. Third, SVM is not suitable when the number of the training data sample is lower than the number of features for each data point. And finally, since the SVM algorithm works by placing data points, there is no probabilistic explanation for the classification above and below the hyperplane classification.

The previous three studies have compared machine learning approaches (e.g., SVM and RF) to build their predictor. However, in the present study, the performance of two neural networks and four different machine learning algorithms (SNN, SVM, KNN, and RF) was compared and selected based on the best predictor of API.

It is essential to define which properties of the aptamer and protein determine their potential for interaction. The lowest performance among datasets was achieved when aptamer features were combined with six protein properties (i.e., hydrophobicity, hydrophilicity, mass, polarity, molecular weight, melting point). According to the^79–86^, energetic and conformational properties have essential effects on protein interactions. Therefore, only the presence of features which are depending on the physicochemical properties and absence of energy and conformational properties could be the reason for low performance.

According to Fig. 2, the k-mer aptamer frequency implies that the k-mer usage is an essential factor for APIs. This finding is justified by the previous studies^87–92^ which proved k-mer frequency plays an important role in interaction related to riboswitch, DNA, RNA, ncRNA, lncRNA, etc. This may be due to aptamers in this study were considered as the type of RNA and DNA.

In the second place, there is feature composition B. As follows, in the third place, there is feature composition C. In the fourth place, other effective traits are feature composition A, composition D, and E, in which their counts are nearly remaining equal. This finding means that the impact of using these three feature composition is the same. And in the last place, there is feature composition F. Since our study targets are proteins, according to previous studies on protein interaction and protein complexes^17,93–99^, it is shown that physicochemical properties (e.g., hydrophobicity, hydrophilicity, mass, volume, etc.) are the main factors which affected on protein interaction. For example, according to^100^, high molecular weight provides strong protein binding affinity. It has also been shown that aptamers are sensitive protein binding based on the local environment polarity at different modification sites^101^. However, the effect of the melting point on protein binding is also indicated^102^.

In this study, we applied RF strategy for feature selection and ranking features. The optimal number of features was set to 193 by several experiments on RF parameters and different numbers of optimal features. The 193 optimal features were selected according to the nature of our dataset and the optimized parameters, which we set to RF strategy. The parameters were set based on our several feature selection experiments.

Roc curves have been broadly used in machine learning and deep learning approaches for performance evaluation^86,103–106^. Therefore, as a popular method for performance evaluation, the ROC curve was utilized in our experiments. Which, ROC instead of considering only the numerical AUC values could be a better strategy in this study.

An oscillation in loss and accuracy in our model can be because of the nature of our dataset. It means that the number of API data (which are the result of laboratory results) that are recorded in freebase and aptagen databases is low (only 850). Since deep learning methods require large volumes of data, therefore, in this study, we see oscillation in loss and accuracy. Indeed, in the future, with more laboratory experiments on API, the amount of API data will be increased, and consequently, the results of deep learning models on them will be better.

## Conclusion

In this study, we have presented AptaNet, a novel deep learning method for predicting API. AptaNet is unique in its exploitation of sequence-based features for aptamers along with the physicochemical and conformational properties for targets to predict API. It also uses a balancing technique and a deep neural network. We have performed extensive experiments to analyze and test AptaNet performance. Experimental evaluations show that, on our 32 benchmark datasets, AptaNet has superior performance compared to other methods examined in this study in terms of accuracy. Moreover, AptaNet has shown to be able to provide biological insights into understanding API's nature, which can be helpful for all aptamer scientists and researchers.

There is still a lot of room for improvement in this field. The study mentioned above focuses on target proteins, but there are other types of targets, such as compounds. Due to the important role of aptamers in various biological processes, further research is needed to focus on the aptamer's interactions with other types of targets. Additionally, due to the large number of properties of aptamers and proteins that can affect the API, further investigations are required to use other features of aptamers and proteins that have been recommended in the literature. Since the existence of an accessible web-server is required in this field, and given the successful results of AptaNet, other extensive efforts are required to provide a powerful web server based on the predicting method presented in this article in the future. Research on aptamer–target interaction prediction is likely to continue for the next years with deep learning approaches and unprecedented feature extraction strategies for aptamers and targets. Consequently, it leads to new opportunities and challenges in this scope.

## Materials and methods

**Figure 5:**
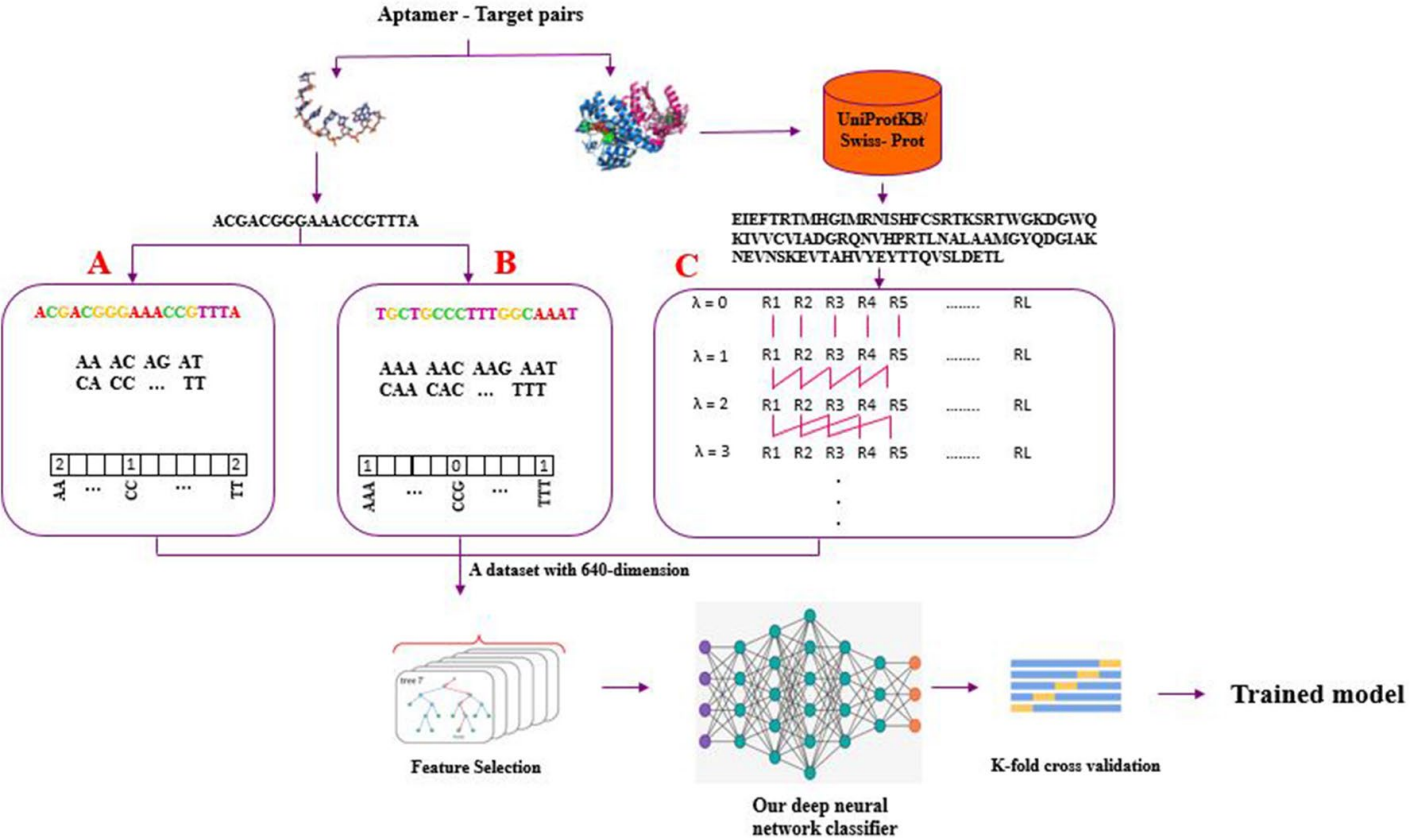
A schematic overview of the training module of AptaNet. For a given sequence (A) Shows K-mer frequency strategy for aptamer encoding; (B) depicts Reverse compliment k-mer strategy for aptamer encoding and (C) Displays Pseudo-Amino Acid Composition strategy for protein encoding. The figure is drawn by using of Microsoft PowerPoint 2016 that is available in https://www.office.com/.

This section provides detailed information of the datasets, feature extraction methods, balancing strategy, two types of examined deep neural networks, feature selection method, and evaluation metrics used in this study. All the methods were implemented in python language using Python 3.6 version. Keras and Scikit-learn library of python was used to implement deep learning methods and the machine learning algorithms, respectively. All experiments were conducted in a Google collaboratory notebook environment. Also, each experiment was carried out five times, and the average of the results was reported. Figure 5 shows the training module of our proposed neural network, AptaNet. The training dataset of AptaNet includes both interacting (positive) and non-interacting (negative) aptamer-protein pairs. For each sample of the aptamer-protein pairs, aptamer sequences were obtained from Aptagen^107^ and aptamer free base^108^ databases.

Similarly, protein sequences are obtained from Swissprot^109^ based on their protein-id. Next, a balancing strategy was applied to prevent the unbalancing problem. Then, feature extraction methods used these data to generate k-mer and Revck-mer for aptamers features and AAC and PseAAC for protein features. After that, by applying a feature selection method, important features were ranked and selected. Finally, obtained features in this step were fed to AptaNet, which learns the model for predicting API.

**Figure 6:**
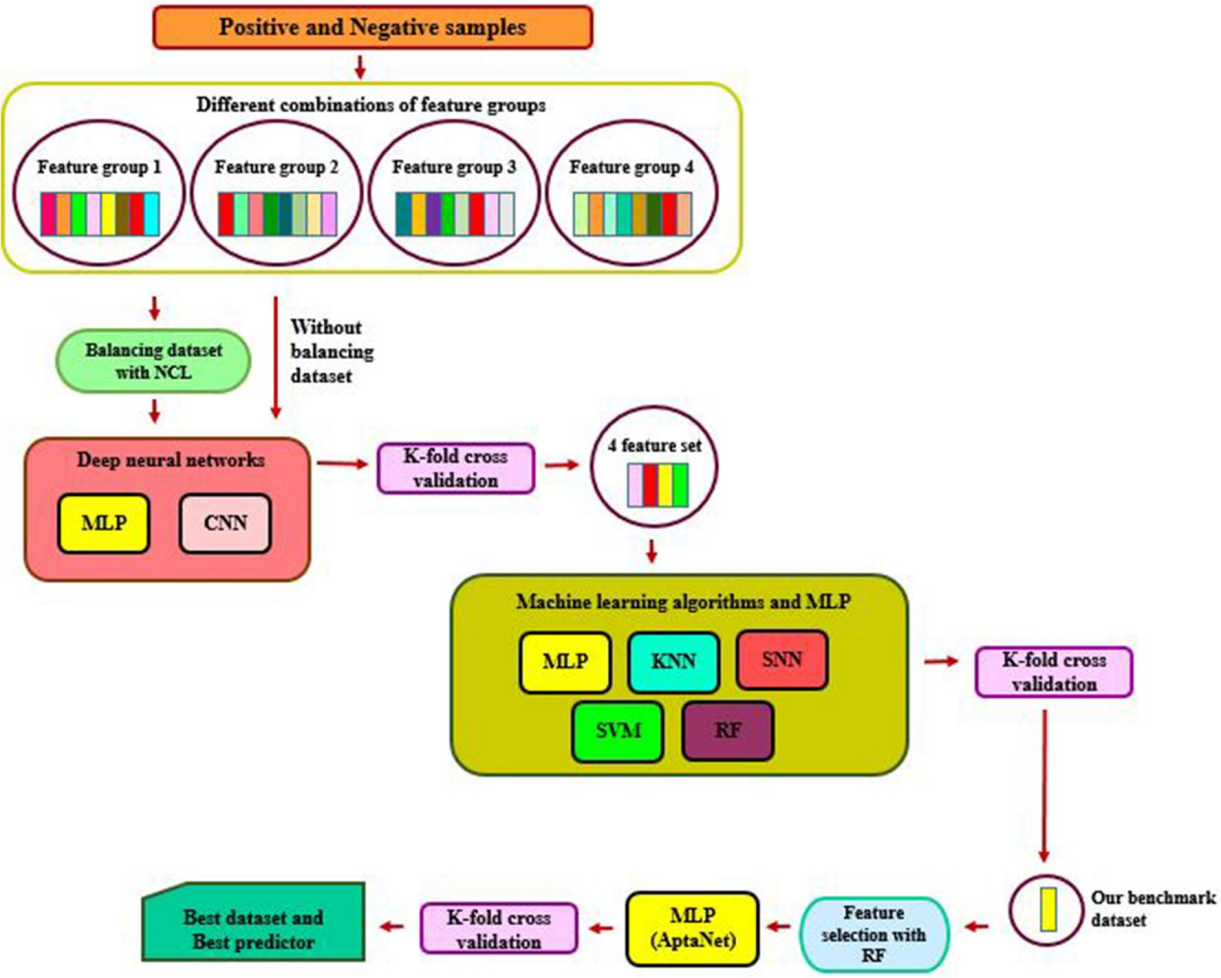
Flowchart of performed methodology to find the best dataset and predictor for potential aptamer-protein prediction. Where feature group 1 contained k-mer (k = 3) frequency for aptamers and 24 physicochemical and conformational properties of proteins sequences; group 2 involved k-mer (k = 4) frequency for aptamers and 24 physicochemical and conformational properties of proteins sequences, group 3 comprised Revckmer (k = 3) frequency for aptamers and 24 physicochemical and conformational properties of proteins sequences and group 4 included Revckmer (k = 4) frequency for aptamers and 24 physicochemical and conformational properties of proteins sequences. *NCL* neighborhood cleaning, *MLP* multi-layer perceptron, *CNN* convolutional neural network, *KNN* k nearest neighbor, *SNN* neural network, *SVM* support vector machine and *RF* random forest. The figure is drawn by using of Microsoft PowerPoint 2016 that is available in https://www.office.com/.

We perform several kinds of experiments. In particular, we performed four different sets of experiments: First, we investigated the effectiveness of the different feature groups, as mentioned in Table 1. We investigated the dataset effectiveness by considering the performance of multi-layer perceptron (MLP) and convolutional neural network (CNN) neural networks on the 32 different datasets. Secondly, we compared the performance of the two deep neural networks used in our research. Subsequently, we compared our model against some machine learning algorithms. Finally, we investigate the result of the feature selection method and AptaNet. In Fig. 6, the overall workflow for our methodology is graphically demonstrated.

### Data collection

To prepare our dataset, we obtained known API from two different databases: *Aptagen* and *Aptamer Base*. Aptagen contains 554 entries of interactions, which among them a total number of 477 are aptamers of RNA/DNA interacting and 241 target proteins. Freebase consists of 1638 interaction entries which 1381 of them are aptamers of DNA/RNA and 211 target proteins. For the proteins, since the identifiers of target proteins are presented in aptagen and Aptamer Base, like *Aspartame*, *Caffeine*, *Colicin E3*, etc., therefore, we prepared their sequences by searching in UniProtKB/Swiss-Prot based on the best name matches. For Aptagen, in 477 aptamers, there is 269 interaction with the 241 protein targets. Therefore, the 269 pairs of APIs are considered as positive samples. And for Aptamer Base in 1381 aptamers, only 725 interaction with 164 proteins was exist, so the 725 API are assumed as positive samples. To remove duplicate APIs between two databases, we unified the IDs of aptamers and the proteins. Thus, 850 APIs with 452 proteins target were obtained. Since all collected APIs are considered as positive instances and negative APIs are not determined in the databases listed. Therefore, negative instances of APIs were generated by a random pairs of aptamers and proteins, so that they did not any overlap with the positive instances. Finally, the total number of instances were 3404, which contain 850 positive and 2554 negative instances.

### Balancing the dataset

Generally, in classification problems, learning methods assume that the number of samples in each class is almost equal^110^. However, in most real conditions, the class distribution is not similar because the number of representations of some classes is much more than the others. As a result, it can restrict the learning model's performance since they will be biased towards the majority class. Therefore, in this study, we used neighborhood cleaning to deal with such incompatibility (NCL). NCL is categorized into the groups of under-sampling strategy^111^. NCL was first introduced by Laurikkala^112^ for balancing dataset by removing some instances from the majority class randomly to reduce the size of the majority class with/ or without replacement.

### NCL procedure

**Figure 7:**
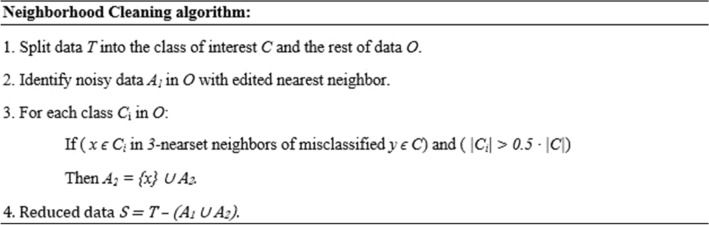
The Neighborhood Cleaning Algorithm. The figure is drawn by using of Microsoft PowerPoint 2016 that is available in https://www.office.com/.

Suppose we have an imbalanced dataset where O is a majority class, and C is a minority class. So, NCL, by using Wilson's edited nearest neighbor rule (ENN)^113^, identified noisy data (A_1_) and reduced O by removing A_1_ in O. In other words, ENN removes instances that differ from at least two of their three nearest neighbors. Furthermore, NCL is intensifying size reduction by removing the three nearest neighbors that misclassify instances of C. Figure 7 describes the NCL algorithm.

### Feature construction

In this study, Kmer frequency and Reverse compliment k-mer (*k* = 2, 3) were adopted separately to encode the aptamer sequences. The amino acid composition and pseudo-amino acid composition were employed to encode the protein sequences.

### K-mer frequency

Since, T-(Thymine) in DNA is similar to U-(uracil) in RNA therefore, we converted each RNA to DNA sequences by replacing U to T. K-mers are subsequences of length *k* (A, T, C, and G) to represent the DNAs sequence. Suppose *n* is the number of possible monomers (A, C, G, and T) so, a total possible k-mers will be *n*^*k*^. (See Fig. 5A) In this study, *k* = 3, 4 were adopted for each aptamer, and as a result, each aptamer was encoded into an 84-dimension for k = 3 and *339*-dimension for k = 4 numerical vector.

### Reverse compliment k-mer

The reverse complement of a DNA sequence is organized by replacing T and A, exchanging G and C, and reversing the letters. **(**See Fig. 5B) As an example, if k = 2 so there are *16* basic k-mers in total, but by reverse compliment k-mers, there are *ten* different k-mers. Therefore, the total possible number of reverse complement k-mer will be calculated as follows:

1 $$\left\{\begin{array}{l}{2}^{2k-1} (k=\mathrm{1,3},5, ...)\\ {2}^{2k-1}+{2}^{k-1 }(k=\mathrm{2,4},6, ...)\end{array}\right.$$

We set (*k* = 3, 4) for each aptamer, and as a result, each aptamer was encoded into a *44*-dimensional vector for k = 3 and a *179*-dimensional vector for k = 4. In this study, to generate characteristics of aptamers, we used a powerful python package known as RepDNA^114^.

### Amino acid composition (AAC)

AAC is a kind of protein sequence feature based on protein attributes, like folding types, secondary structure, domain, subcellular location, etc. AAC calculates the frequency of each amino acid in a protein sequence. For a protein sequence with *N* amino acid residues, the frequencies of each residue could be considered as follows:

2 $$ f\left( t \right) = \frac{N\left( t \right)}{N}, t \epsilon  $$

*N(t)* is the number of amino acid type *t*.

### Pseudo-amino acid composition (PAAC)

Chou first introduced this group of descriptors for the prediction of protein subcellular properties^115^. (See Fig. 5C) PseAAC has been used as an effective feature extraction method in several biological problems^31–37^. The PseAAC method can be demonstrated as defined below:

Given a protein chain P with N amino acid residues:

3 $${P=R}_{1}{R}_{2}{R}_{3}\dots {R}_{N}$$

The protein sequence order effect can be demonstrated by a set of separate correlation factors, as follows:

4 $$\left\{\begin{array}{l}{\theta }_{1}=\frac{1}{L-1}\sum_{i=1}^{L-1}\Theta ({R}_{i}, {R}_{i+1})\\ {\theta }_{2}=\frac{1}{L-2}\sum_{i=1}^{L-2}\Theta ({R}_{i}, {R}_{i+2})\\ {\theta }_{3}=\frac{1}{L-3}\sum_{i=1}^{L-3}\Theta \left({R}_{i}, {R}_{i+3}\right)\\ .\\ .\\ .\\ {\theta }_{\lambda }=\frac{1}{L-\lambda }\sum_{i=1}^{L-\lambda }\Theta \left({R}_{i}, {R}_{i+\lambda }\right), (\lambda <L)\end{array}\right.$$

where θ_1_, θ_2_, …, θ_λ_ are called the 1-tier, 2-tier, and λ-th tier correlation factors, respectively. The correlation function can be shown by:

5 $$\Theta \left({R}_{i}, {R}_{j}\right)= \frac{1}{3}\left\{{\left[{H}_{1}\left({R}_{j}\right)-{H}_{1}\left({R}_{i}\right)\right]}^{2}+{\left[{H}_{2}\left({R}_{j}\right)-{H}_{2}\left({R}_{i}\right)\right]}^{2}+{\left[M\left({R}_{j}\right)-M\left({R}_{i}\right)\right]}^{2}\right\}$$

where H_1_(R_i_), H_2_(R_i_), and M(R_i_) are, some properties (e.g., physicochemical, conformational, and energetic) value for the amino acid R_i_; and also H_1_(R_j_), H_2_(R_j_), and M(R_j_) are the corresponding values of the amino acid R_j_. Notably, each property values are transformed from the original values based on the following equation:

6 $$\left\{\begin{array}{c}{H}_{1}\left(i\right)=\frac{{H}_{1}^{0}\left(i\right)-\sum_{i=1}^{20}\frac{{H}_{1}^{0}\left(i\right)}{20}}{\sqrt{\frac{\sum_{i=1}^{20}\left[{H}_{1}^{0}\left(i\right)-\sum_{i=1}^{20}\frac{{H}_{1}^{0}\left(i\right)}{20}\right]}{20}}}\\ {H}_{2}\left(i\right)=\frac{{H}_{2}^{0}\left(i\right)-\sum_{i=1}^{20}\frac{{H}_{2}^{0}\left(i\right)}{20}}{\sqrt{\frac{\sum_{i=1}^{20}\left[{H}_{2}^{0}\left(i\right)-\sum_{i=1}^{20}\frac{{H}_{2}^{0}\left(i\right)}{20}\right]}{20}}} \\ M\left(i\right)=\frac{{M}^{0}\left(i\right)-\sum_{i=1}^{20}\frac{{M}^{0}\left(i\right)}{20}}{\sqrt{\frac{\sum_{i=1}^{20}\left[{M}^{0}\left(i\right)-\sum_{i=1}^{20}\frac{{M}^{0}\left(i\right)}{20}\right]}{20}}}\end{array}\right.$$

where H_1_ (i), H_2_ (i), and M (i) are the original property values for the amino acids. Therefore, for a protein sequence P, the PseAAC can be illustrated by a vector with (20 + $\lambda$)—Dimensional as:

7 $${\left[{V}_{1},{V}_{2}, \dots ,{V}_{20}, {V}_{21}, \dots , {V}_{20+\lambda } \right]}^{T}$$

T is the transpose operator.

8 $${X}_{u}=\left\{\begin{array}{l}\frac{{f}_{u}}{\sum_{i=1}^{20}{f}_{i}+\omega \sum_{j=1}^{\lambda }{\theta }_{j}}, (1\le u\le 20)\\ \frac{{\omega \theta }_{u-20}}{\sum_{i=1}^{20}{f}_{i}+\omega \sum_{j=1}^{\lambda }{\theta }_{j}}, (20+1\le u\le 20+\lambda )\end{array}\right.$$

where for the mentioned protein sequence P, f_i_ shows the occurrence frequencies of the 20 amino acids, and θj shows j-th tier sequence correlation factor that is calculated based on Eq. (2), and ω shows the weight factor of the sequence order effect. We set ω = 0.05. According to the above description, the first 20 components in Eq. (5) shows the amino acid composition effect, and the other remaining components (20 + 1 to 20 + λ) show the effect of sequence order. So, the whole of 20 + λ components will be PseAAC. We set λ = 30.

In this study, we used 24 physicochemical and biochemical (i.e., hydrophobicity, hydrophilicity, mass, polarity, molecular weight, melting point, transfer-free energy, buriability, bulkiness, solvation free energy, relative mutability, residue volume, volume, amino acid distribution, hydration number, isoelectric point, compressibility, chromatographic index, unfolding entropy change, unfolding enthalpy, unfolding Gibbs free energy change, power to beat the N terminal, C terminal and middle of alpha helix) properties of amino acids. The 24 properties were retrieved from^116,117^ which could be found in Supplementary Table S3.

Also, in our study, to generate characteristics of proteins, we used a python package known as iFeature^118^.

### Description of deep neural network model

#### Multi-layer perceptron (MLP)

We have selected the MLP as our classification model. A seven-layer neuron network was performed in the fully connected layer to generate the final prediction of interaction between aptamer and protein. The number of layers we selected here was depended on the various tests among the seven different layers (i.e., *3, 4, 5, 6, 7, 8*, and *9*) and the comparison of their outcomes. Finally, the best outcomes were obtained when the seven-layer network was applied.

All the neuron units in each layer (layer i) were connected to its previous layer (i − 1), and outcomes produced by using non-linear transformation function f as follows:

9 $${o}_{j}=\left(\sum_{i=1}^{H}{w}_{i}{o}_{i}+{b}_{i}\right)$$

where H represents the number of hidden neurons, w and b are the weights and bias of neuron j, which summarize all the hidden units. After each fully-connected layer, the network performed an activation function named rectified linear unit (Relu).

10 $$ReLU\left(x\right)=\left\{\begin{array}{c}x, x\ge 0\\ 0, x<0\end{array}\right.$$

Relu is a non-linear function that can extract hidden patterns in the data and reduce gradient vanishing. The Dropout was applied in order to avoid overfitting behind every fully connected layer. The outcome in the last layer was obtained using the sigmoid function as follows:

11 $$Sigmoid\left(x\right)=\frac{1}{1+{e}^{-x}}$$

To train the network, we minimized the objective function for loss minimization. We used a function named binary cross-entropy cost function C, as follows:

12 $$C= -\frac{1}{n}\sum_{x}\sum_{t}\left[yIna+(1-y)In(1-a)\right]$$

where C is the output of the loss function called the binary cross-entropy cost function. Furthermore, x indicates the training sample index, and t represents the index of different labels, y indicates the true value of sample x, which can be *0* or *1*. And *a* is the predicted output of the network for *0* or *1* value given input sample x. When the predicted outputs are close to the true values, the value of C will get less. Since the cross-entropy is a non-negative function, so to get the best prediction, the function must minimize.

### Comparison with machine learning algorithms

To compare our model and some machine learning algorithms, we compared the performance of AptaNet against some machine learning algorithms. We selected five machine learning algorithms, namely, SNN, KNN, RF, and SVM. We performed fivefold cross-validation to evaluate the performance of the test and training set.

### Feature selection

In order to prevent overfitting and select the most important features, we used the RF algorithm. RF is one of the robust machine learning algorithms created by Loe Breiman^119^. RF is an ensemble learning containing multiple decision trees. Each of the decision trees is created based on a random extraction of the instances and the features. Since each tree does not check all features and instances, so, it can be concluded that trees are de-correlated, and therefore the possibility of their over-fitting is less.

RF selects important features by ranking all features based on the improvement of the node purity. The node's probability is the number of instances that reach the node, divided by the whole number of instances. Therefore, the importance of the features has a direct relation with the high value of the probability. We chose forests containing nine trees based on our feature selection experiments.

### Performance evaluation

In this study, we used fivefold cross-validation to evaluate the performance of our model. During this procedure, the whole dataset is evenly and randomly divided into five folds which four folds are used for training and one for testing. This method was repeated five times, and each instance was tested just once. In order of evaluation of the predictor performance, the prediction accuracy, f1_macro, precision, specificity, sensitivity (recall), and matthews's correlation coefficient were computed as below:

13 $$Accuracy=\frac{TP+TN}{TP+TN+FP+FN}$$

14 $$Precision= \frac{TP}{TP+FP}$$

15 $$F1 score= \frac{Precision\times Sensitivity}{Precision+Sensitivity}$$

16 $$Sensitivity= \frac{TP}{TP+FN}$$

17 $$Specificity= \frac{TN}{TN+FP}$$

18 $$Matthews{^{\prime}}s Correlation Coefficient= \frac{(TP\times TN)-(FP \times FN)}{\surd (TP+FP)(TP+FN)(TN+FP)(TN+FN)}$$

where TP, FP, TN, and FN represent true positive, false positive, true negative, and false negative, respectively.

## Supplementary Information

Supplementary Table S1.

Supplementary Table S2.

Supplementary Table S3.
